# A novel *CLCNKB* mutation in a Chinese girl with classic Bartter syndrome: a case report

**DOI:** 10.1186/s12881-019-0869-9

**Published:** 2019-08-13

**Authors:** Binlu Zhu, Hong Jiang, Meiling Cao, Xueqi Zhao, Hongkun Jiang

**Affiliations:** Department of Pediatrics, The First Hospital of China Medical University, No. 155 Nanjing North Street, Heping District, Shenyang, 110001 Liaoning Province People’s Republic of China

**Keywords:** Bartter syndrome, *CLCNKB*, Growth hormone deficiency, Proteinuria, Atrial septal defect

## Abstract

**Background:**

Bartter syndrome (BS) is a rare autosomal recessive disorder of salt reabsorption at the thick ascending limb of the Henle loop, characterized by hypokalemia, salt loss, metabolic alkalosis, hyperreninemic hyperaldosteronism with normal blood pressure. BS type III, often known as classic BS (CBS), is caused by loss-of-function mutations in *CLCNKB* (*chloride voltage-gated channel Kb*) encoding basolateral ClC-Kb.

**Case presentation:**

We reported a 15-year-old CBS patient with a compound heterozygous mutation of *CLCNKB* gene. She first presented with vomiting, hypokalemic metabolic alkalosis at the age of 4 months, and was clinically diagnosed as CBS. Indomethacin, spironolactone and oral potassium were started from then. During follow-up, the serum electrolyte levels were generally normal, but the patient showed failure to thrive and growth hormone (GH) deficiency was diagnosed. The recombinant human GH therapy was performed, and the growth velocity was improved. When she was 14, severe proteinuria and chronic kidney disease (CKD) were developed. Renal biopsy showed focal segmental glomerulosclerosis (FSGS) with juxtaglomerular apparatus cell hyperplasia, and genetic testing revealed a point deletion of c.1696delG (p. Glu566fs) and a fragment deletion of exon 2–3 deletions in *CLCNKB* gene. Apart from the CBS, ostium secundum atrial septal defect (ASD) was diagnosed by echocardiography.

**Conclusions:**

This is the first report of this compound heterozygous of *CLCNKB* gene in BS Children. Our findings contribute to a growing list of *CLCNKB* mutations associated with CBS. Some recessive mutations can induce CBS in combination with other mutations.

## Background

Bartter syndrome (BS) and Gitelman syndrome (GS) are rare autosomal salt-losing tubulopathies, characterized by hypokalemic metabolic alkalosis, hyperreninemic hyperaldosteronism with normal blood pressure and juxtaglomerular apparatus cell hyperplasia [1]. BS is clinically categorized as antenatal BS (ABS) and classic BS (CBS); BS is also categorized into five genetic subtypes based on the underlying mutant gene: *SLC12A1* gene encoding the sodium-potassium-chloride cotransporter NKCC2 for type I (OMIM #601678); *KCNJ1* gene encoding the apical inwardly rectifying potassium channel ROMK for type II (OMIM #241200); *CLCNKB* (*chloride voltage-gated channel Kb*) gene encoding the basolateral chloride channel ClC-Kb for type III (OMIM #607364); *BSND* gene encoding the β-subunit for ClC-Ka and ClC-Kb for type IVa (OMIM #602522) with sensorineural deafness; *CLCNKB* and *CLCNKA* co-mutated for type IVb (OMIM #613090); *CASR* gene encoding the basolateral calcium sensing receptor for type V (OMIM #601199) [2]. BS Type III, often known as CBS, is characterized by salt wasting from the renal tubules, mainly the thick ascending limb of the Henle loop [3]. CBS should be differentiated with GS (OMIM #263800), GS is a milder disease frequently associated with hypomagnesemia and hypocalciuria, caused by dysfunction of *SLC12A3* gene encoding the sodium chloride co-transporter NCCT in the distal convoluted tubule [4].

Patients with CBS fail to thrive from infancy or early childhood and exhibit hypokalemia, metabolic alkalosis, polyuria, polydipsia, volume contraction, muscle weakness, growth retardation and nephrocalcinosis. Recently, growth hormone (GH) deficiency has been reported in a few children with BS or GS [5–7]. However, a clear pathogenesis of growth failure has not been elucidated yet. In addition, there are also limited numbers of patients with BS or GS who had proteinuria associated with focal segmental glomerulosclerosis (FSGS) in the literature [8–10].

We reported a unique case of CBS associated with GH deficiency and atrial septal defect (ASD) with a novel compound heterozygous mutation in the *CLCNKB* gene.

## Case presentation

**Fig. 1:**
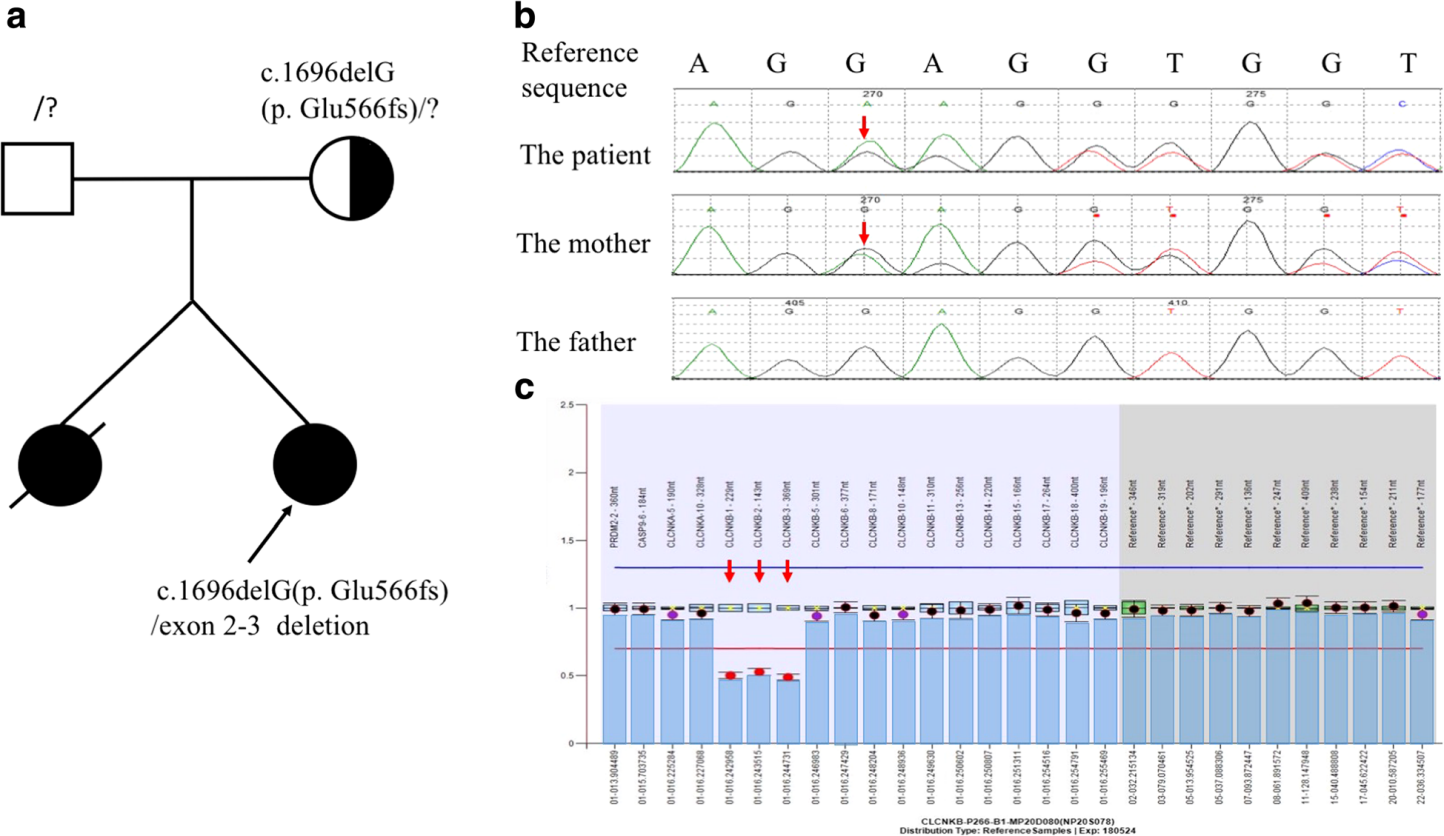
Mutation analysis by direct sequencing in *CLCNKB*. **a** pedigree of the patient’s family. The arrow indicates the proband; her elder identical twin sister was clinically hypothesized died of BS. **b** Mutation analysis by direct generation sequencing in *CLCNKB*. The patient is compound heterozygous, the point deletion of c.1696delG (p. Glu566fs) inherited from her mother. **c** MLPA showed the other heterozygous mutation of the deletion of exon 2–3 in the *CLCNKB* of the patient. (Arrow shows the position of the mutation)

The patient (Fig. 1) was a 15-year-old Chinese girl. She was born as the younger one of twins at 38 weeks gestational age by planned caesarean section delivery, with a birth weight of 2.3 kg and length of 46 cm, and the 1,5 min Apgar scores were 10. There was no consanguinity between parents. Her elder identical twin sister was clinically hypothesized died of BS at the age of 6 months. Other family members had no histories of hereditary diseases. At 4 months old, she was transferred to a tertiary referral center as she presented with frequent vomiting, dehydration, hypokalemia and concomitant metabolic alkalosis. Plasma renin and aldosterone were markedly elevated, while blood pressure was within the normal range. She was clinically diagnosed with CBS. Oral Spironolactone, indomethacin and potassium supplements were started. During follow-up, despite the appropriate therapy and generally normalized serum electrolyte, the girl showed failure to thrive. At the age of 6 years, her height was 97 cm(<3rd percentile) and weight was 13 kg(<3rd percentile). There was no abnormality in renal ultrasonography and magnetic resonance imaging of pituitary gland. GH stimulation tests revealed GH deficiency, and recombinant human GH replacement therapy (0.1 IU/kg per day) was started (Table 1). After 6 years of treatment, the annual increase in her length had reached 11 cm on average. Ostium secundum type ASD was diagnosed by echocardiography. Proteinuria was first indicated when she was 12 years old from the results of a urinalysis during the follow-up but had not been noticed.

**Table 1:** Clinical and laboratory data and treatment of the patient during follow-up

Age, years	6	7	8	9	10	11	12	13	14
Height, cm (percentile)	97(<3)	111(<3)	119 (3)	126 (10)	132 (10)	143 (25)	150 (25–50)	153 (25–50)	155 (25–50)
Weight, kg (percentile) Serum	13(<3)	18 (3)	22 (25)	23 (10)	26 (10)	34 (25–50)	41 (50)	42 (25–50)	45 (25–50)
BUN, mmol/l (1.7–8.3) ^a^	2.42	2.26	3.73	6.45	4.27	5.32	4.93	6.41	13.49
Cr, umol/l (53–106) ^a^	40.9	48.1	44.3	54.4	56.1	59.2	57	70.9	175
Na, mmol/l (136–146) ^a^	136.2	135.3	136.6	145.4	145.9	139.2	145.1	146.1	145
K, mmol/l (3.5–5.5) ^a^	3.78	3.79	3.35	3.64	3.49	3.57	3.12	3.79	2.99
Cl, mmol/l (97–107) ^a^	92.7	93.3	92.3	103.6	99.5	95.1	105.6	103.5	102.3
HCO3^−^, mmol/l (21–29) ^a^ Treatment	33.9	30.6	30.6	29.3	26.8	29.7	24.1	30.2	25.4
recombinant human GH, IU/kg/d	0.1	0.1	0.1	0.15	0.15	0.15	–	–	–

*BUN* blood urea nitrogen, *Cr* creatinine, *Na* sodium, *K* potassium, *Cl* chloride, *HCO3*^*−*^ bicarbonate

^a^Normal values in parentheses

At 14 years, serum creatinine and blood urea nitrogen levels were elevated and she was admitted to our hospital for further evaluation of renal function. On physical examination, her height was 155 cm, body weight was 45 kg, blood pressure was 120/74 mmHg, cardiac auscultation revealed a grade 3/6 systolic blowing murmur at the second and the third left intercostal space. Biochemical analyses showed normal serum pH (7.45) and normal levels of blood sodium, chloride, bicarbonate (HCO_3_^−^), calcium, phosphorus and magnesium. However, serum potassium was low (2.99 mmol/L, reference range: 3.5–5.3 mmol/L). The plasma renin activity and AngiotensinII were high both in decubitus (plasma renin activity 1.5 ng/ml and AngiotensinII 149.58 ng/ml; reference value 0.5–0.79 ng/ml and 28.2–52.3 ng/ml) and upright position (plasma renin activity 8.67 ng/ml and AngiotensinII 149.58 ng/ml; reference value 0.93–6.56 ng/ml and 55.3–115.3 ng/ml). She had moderate renal dysfunction [BUN 13.49 mmol/L; Cr 175 umol/L (19.79 mg/dl); 24-h creatinine clearance 43 ml/min per 1.73 m^2^ body surface area, indicating moderate CKD (Grade 3b) (2012 KDIGO guidelines)], severe proteinuria (urinary protein 8.861 g/day, serum total protein 54.2 g/L; reference value 65–85 g/L, serum albumin 30.9 g/L; reference value 40–55 g/L, urine β_2_-microglobulin 3.16 mg/L; reference value < 0.23 mg/L) and normal urine calcium excretion (0.11 mmol/L). Neither nephrocalcinosis nor nephrolithiasis was detected by renal ultrasonography. However, renal dynamic imaging (scintigraphy with 99mTc-DTPA) revealed glomerular filtration rate remarkably decreased [total glomerular filtration rate (GFR) about 49.7 mL/min per 1.73m^2^, left GFR about 26.9 mL/min, right GFR about 22.9 mL/min]. The transthoracic echocardiography revealed a 22-27 mm secundum atrial septal defect with left-to-right shunt. While the left ventricular ejection fraction (57%) and diastolic function were normal, the left ventricular volumes decreased (left ventricular end-diastolic volume:48 ml, left ventricular end-systolic volume:20 ml). Electrocardiogram was normal.

### Genetic analysis and results

After obtaining the informed consents from the patient and her parents, direct sequencing of known BS genes was performed. The sequencing procedure were performed by KingMed Diagnostics Test Laboratory (Shenyang, China) which provides the third-party inspection services. While the genetic studies for *SLC12A1*, *KCNJ1*, *BSND*, *CASR* and *SLC12A3* were all negative, two novel compound mutations in *CLCNKB* were detected. The results showed one is a heterozygous mutation c.1696delG in exon 16 of *CLCNKB*, resulting in p. Glu566fs amino acid frameshift mutation. The one inherited from her mother. The other one is a heterozygous deletion of exon 2–3, which was confirmed by multiplex ligation-dependent probe amplification (MLPA) of *CLCNKB* (Fig. 1). Neither of these two mutations have been described before or detected in 100 control samples (reference sequence: NM_000085.4). Because the predicted devastating effect on protein structure of the 2 alleles and the patients’ clinical features, we speculate these mutations are pathogenetic.

### Renal pathology findings

**Fig. 2:**
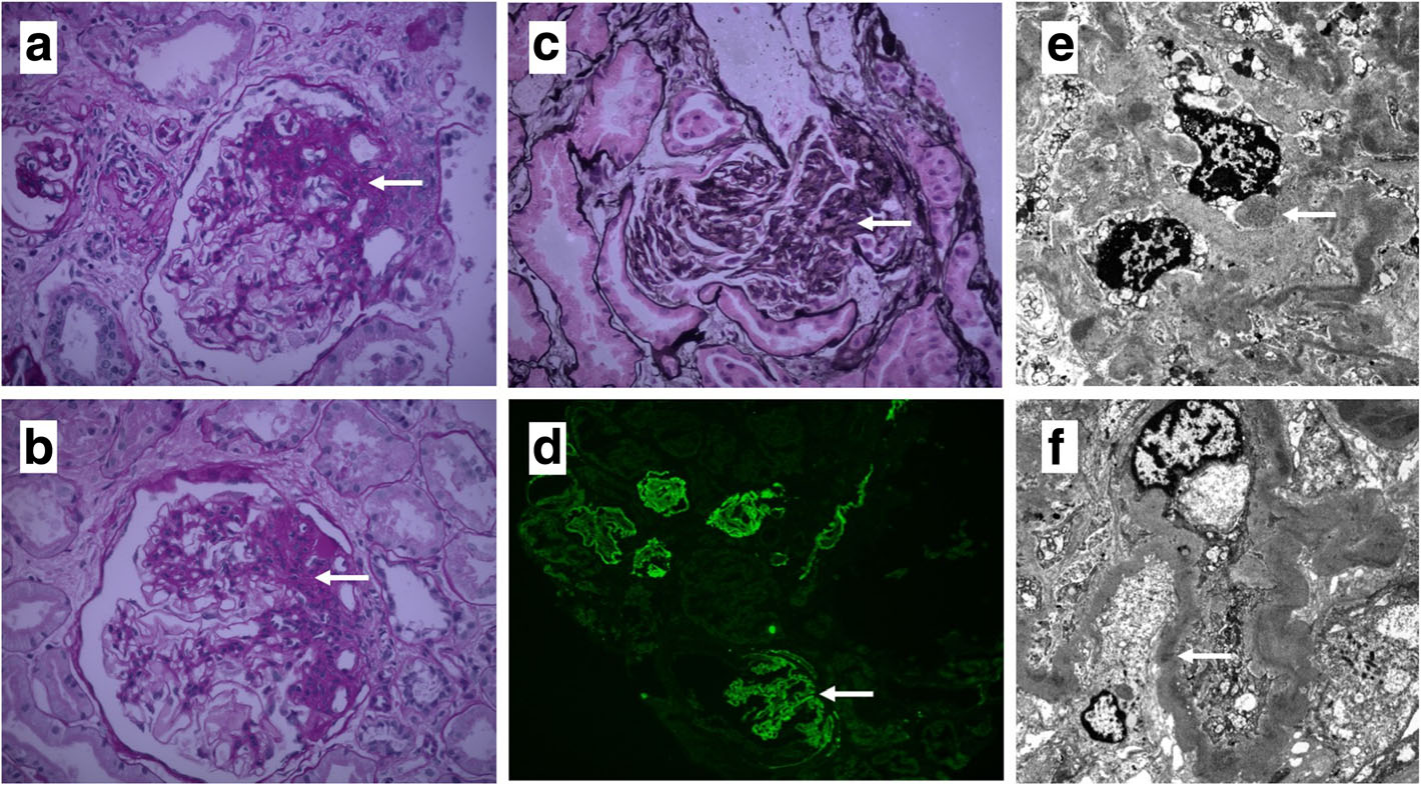
Renal biopsy in a patient with CBS. Photomicrograph of renal biopsy specimen with HE, PAS (**a, b**) stain showed mesangial cell and matrix proliferation and PASM stain (**c**) showed focal segmental glomerulosclerosis. The immunofluorescence examination showed Immunoglobulins (IgM +, IgA +/−) and complements (C3 +/−) deposited in the mesangium and capillary wall (**d**). Electron microscopy showed focal segmental glomerulosclerosis with glomerular basement membrane thickened, immune complex deposits in mesangial matrix, vacuolar degeneration of tubular epithelial cells, renal interstitial fibrosis and inflammatory cells infiltration appears (**e**, **f**). (Arrows show the specific features.) (**a**, **b**, **c** and **d** 40× magnification; **e**, **f** 4000× magnification)

Because of the patient’s severe proteinuria, a percutaneous renal biopsy was performed and 17/26 of the results showed glomeruli revealed glomerulosclerosis, 8/26 of the glomeruli revealed FSGS which were located near the vascular pole, the other one was slightly enlarged with mildly increased mesangial cellularity. The microscopic examination of renal tissue showed hyperplasia of cells at the juxtaglomerular apparatus, focal tubular atrophy involving approximately 25% of the cortex, tubulointerstitial fibrosis with infiltration of inflammatory cells and a few foam cells were presented, vascular wall without obvious pathological changes. These findings are compatible with renal histology findings for BS. The immunofluorescence examination of 2/26 of the glomeruli demonstrated dominant granular staining for immunoglobulins (IgM +, IgA +/−) and complements (C3 +/−) in the mesangium and capillary wall. Staining for C1q was negative. Electron microscopy of one sclerotic glomeruli revealed glomerular basement membrane thickened, immune complex deposited in mesangial matrix, vacuolar degeneration of tubular epithelial cells, renal interstitial fibrosis and inflammatory cells infiltration appears (Fig. 2).

## Discussion and conclusions

Type III BS is caused by the mutation in *CLCNKB* gene mapped in chromosome 1p36.13 which encodes a voltage-gated chloride channel protein called ClC-Kb. ClC-Kb is a member of the CIC chloride channel family, which is expressed in the thick ascending limb of Henle’s loop, distal convoluted tubule and cortical collecting tubule and regulates the tubular reabsorption of chloride in the kidney [11]. As a result, mutations inactivate ClC-Kb, reducing chloride as well as sodium reabsorption in the renal tubules. Moreover, the loss of sodium chloride and water activates the renin-angiotensin-aldosterone system, which contributes to the loss of potassium and renal fibrosis [11, 12].

**Fig. 3:**
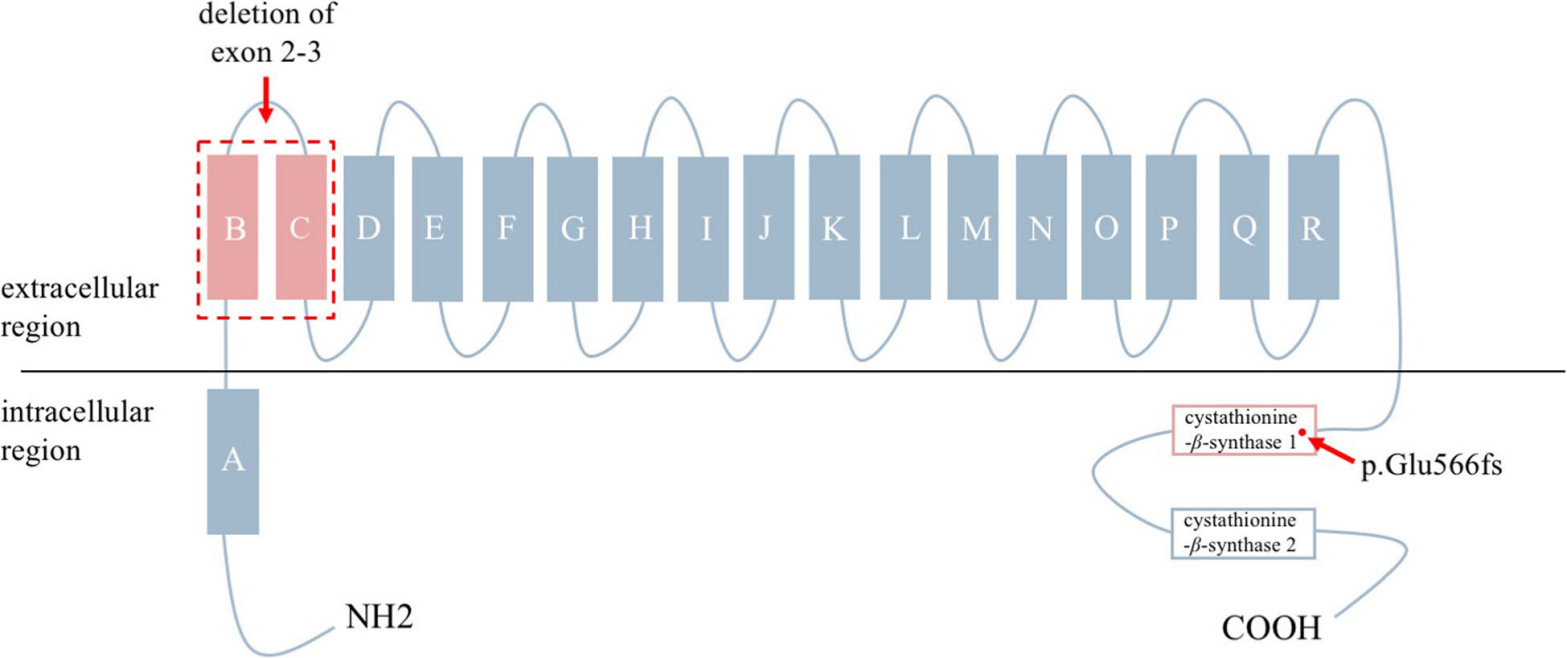
The schematic figure of the ClC-Kb protein. ClC-Kb is a transmembrane protein consisting of 18 α-helices (A to R) and 2 cystathionine-β-synthase domains. The α-helices involved in the selectivity filter, those interacting with Barttin, and those located at the dimer interface. The mutation of the deletion of exon 2–3 is located in α-helix B and C of ClC-Kb, involved in the dimer interface; and p. Glu566fs is located in the cystathionine-β-synthase 1 domain involved in channel common gating and trafficking. These mutations were predicted to result in the production of unstable mRNAs or truncated or absent proteins

In our patient, we identified two different heterozygous *CLCNKB* mutations, neither of the variants has been reported. One was a small deletion c.1696delG in exon 16, which led to the premature termination at codon 571(p.Glu566Argfs*6), leading to a truncated protein. It is located in the same site of another variant p.Arg595Ter from a published case with BS, which is present in one of the cystathionine-β-synthase domains involved in channel common gating and trafficking may decrease or abolish normalized conductance of ClC-Kb [13]. The other one was deletion of exon 2–3, which was confirmed by MLPA. It is located in the junction of the α-helices B and C and the following extracellular region of the ClC-Kb. It probably also be damaging because these large deletions may remove one or more splice sites from ClC-Kb transcript resulted in the production of seriously truncated non-functional protein, however further research is needed to confirm its pathogenicity (Fig. 3). Because her parents declined our suggestion of performing MLPA, we do not clearly confirm whether this mutation was inherited from the patient’s father or occurred de novo. Interestingly, our patient had an elder identical twin sister, who was clinically hypothesized died of BS at 6 months. Although there is no genetic diagnosis, we speculate that genetic effects play an important role in the pathogenesis of the identical twins. Severe (large deletions, frameshift, nonsense, and essential splicing) and missense mutations resulting in poor residual conductance were associated with younger age at diagnosis [13]. We speculate that these compound heterozygous mutations may cause loss-of-function of *CLCNKB* gene associated with the earlier onset of CBS in our patient.

Growth retardation is a common clinical manifestation in children with BS. The underlying pathogenesis of growth retardation in BS is not clearly, but experimental study has shown hypokalemia may be a causative factor of GH deficiency [5]. Rats on a diet poor in potassium exhibit significant reduction with low levels of serum GH and insulin-like growth factor 1, suggested that potassium depletion could have a negative effect on pituitary GH secretion [14, 15]. Although hypokalemia can play a key role in growth retardation in hypokalemic disorders such as BS, some patients still have growth problems after the normalization of serum electrolytes. Based on the literature and our case, we suggest that children with BS or GS may experience growth retardation due to GH deficiency. As in our case, the patient exhibited markedly height gain after recombinant human GH treatment and oral potassium supplements. Thus, GH treatment as well as the correction of serum potassium level is important for optimal growth. Moreover, CKD may alter GH metabolism and organ resistance to GH which as major contributors to growth retardation [16]. Future studies are required for the analysis of the detailed mechanisms of GH deficiency in patients with BS.

The other interesting point in our patient was the presence of CKD (eGFR 43 ml/min per 1.73 m2, Grade 3b) with nephrotic range proteinuria. Renal biopsies of our patient showed FSGS as well as juxtaglomerular apparatus hyperplasia, interstitial fibrosis, as expected in BS and GS. Besides, dominant immunoglobulins (IgM +, IgA +/−) staining along with complements (C3 +/−) was demonstrated in the mesangium and capillary wall, which correlated with scattered electron dense mesangial deposits demonstrated by electron microscopy. These are several possible explanations for pathogenetic mechanisms of the changes BS patient kidneys. One possibility is that chronic stimulation of the renin-angiotensin-aldosterone system, which increased AngiotensinIIin response to chronic renal dysfunction due to salt-losing nephropathy [3, 17]. Another point to consider is that prolonged hypokalemia can lead to hypertrophy and renal fibrosis through activation of transforming growth factor β [18]. Moreover, other studies suggested long-term treatment with nonsteroidal anti-inflammatory drugs and prematurity are increased risk factors for CKD [19, 20]. After discharge, our patient was treated with orally administered potassium supplements, indomethacin and spironolactone. The patient’s creatinine clearance and proteinuria showed marked improvement with these treatments. The mechanism of CKD development is multifactorial, integrated control of serum electrolyte level, angiotensin-converting enzyme inhibitor (indomethacin) and aldosterone antagonist (spironolactone) application are key to reduce and delay patients with chronic renal failure in long-term follow-up. Nonetheless, a better understanding of the mutated proteins will contribute to targeted treatment in BS. Correcting deficiencies in mutated proteins and targeting treatment on mutant gene will shed new light on new therapy.

Furthermore, echocardiography showed that our patient had ASD, but she did not have any clinical manifestations of heart disease. An experimental study has shown that altered transcript regulation of CLC chloride channels does not contribute to the cardiac pathology in different cardiovascular diseases, and it was not shown in congenital heart disease [21]. It is probable that mutations of heart factor genes can cause ASD, detailed genetic analysis is required for definitive diagnosis.

In summary, we report a patient with BS type III who showed CKD with severe proteinuria and growth retardation. Kidney biopsy have shown juxtaglomerular apparatus hyperplasia, interstitial fibrosis and immune complex deposited which were mostly compatible with BS. Diagnosis of CBS was confirmed by the mutation in *CLCNKB* gene. To our knowledge, this is the first time that such a compound heterozygous mutation has been reported in *CLCNKB* gene. This case shows the importance of genetic analysis combined with renal biopsy and clinic laboratory findings in diagnosis and differential diagnosis of CBS. Further study of the molecular mechanism of the gene mutation could possibly provide targets for specific treatment in BS cases.

## Data Availability

The data of the current study are available from the corresponding author on reasonable request.

## Abbreviations

ABS: Antenatal Bartter syndrome
ASD: Atrial septal defect
BS: Bartter syndrome
CBS: Classic Bartter syndrome
CKD: Chronic kidney disease
CLCNKB: Chloride voltage-gated channel Kb
FSGS: Focal segmental glomerulosclerosis
GFR: Glomerular filtration rate
GH: Growth hormone
GS: Gitelman syndrome
MLPA: Multiplex ligation-dependent probe amplification
